# Mitochondrial genome of *Sabella spallanzanii* (Gmelin, 1791) (Sabellida: Sabellidae)

**DOI:** 10.1080/23802359.2021.1872431

**Published:** 2021-02-11

**Authors:** Guillemine Daffe, Yanan Sun, Shane T. Ahyong, Elena K. Kupriyanova

**Affiliations:** a Université de Bordeaux, CNRS, INRAE, Université La Rochelle, Pessac, France; b Australian Museum Research Institute, Australian Museum, Sydney, Australia; c Division of Life Science, The Hong Kong University of Science and Technology, Hong Kong, China; d School of Biological, Earth and Environmental Sciences, University of New South Wales, Kensington, Australia; e Department of Biological Sciences, Macquarie University, Sydney, Australia

**Keywords:** Annelida, Polychaeta, mitogenome

## Abstract

We report the mitochondrial genome of *Sabella spallanzanii*, an invasive Mediterranean sabellid introduced to Australia and New Zealand. The mitogenome is 15,581 bp long and consists of 38 genes, including 13 protein coding genes, two rRNA genes, and 23 tRNA genes. It shows deviations from the putative annelid ground pattern, such as gene order re-arrangements and regions encoding on the negative strand. It is, however, very different from the mitogenome of the closely related serpulid, *Spirobranchus giganteus*. Phylogenetic analyses of the mitochondrial genes support a sister relationship of *Sabella spallanzanii* and *Spirobranchus giganteus*.

*Sabella spallanzanii* (Gmelin 1791) (Sabellidae) is a feather duster worm, native to the Mediterranean, where it forms aggregations in natural habitats and on artificial structures. The species is a highly invasive pest in Australia and New Zealand (Read et al. 2011; Ahyong et al. 2017). Since the first viable population was observed in Western Australia in 1965 (Clapin and Evans 1995), *Sa. spallanzanii* has established along the southern coast of Australia (Murray and Keable 2013). As one of three families within the sedentarian order, Sabellida (Sabellidae, Serpulidae and Fabriciidae), Sabellidae is sister to Serpulidae (calcareous tube worms), which together form a clade as sister to Fabriciidae (Tilic et al. 2020a). Herein we present the first mitochondrial genome for Sabellidae, which represents one of the suspension feeding lineages in Sedentaria. The only other mitochondrial genomes available for Sabellida are the Christmas tree worm *Spirobranchus giganteus* (Pallas 1766) (Serpulidae) and the freshwater *Manayunkia occidentalis* Atkinson, Bartholomew & Rouse, 2020 (Fabriciidae).

The studied specimen was collected from Western Australia (32°12′S, 115°40′E) and deposited in the Australian Museum, Sydney (W. 48385, Collection Manager Dr Stephen Keable, Stephen.Keable@Australian.Museum). Total genomic DNA was isolated from the posterior end of the worm with the DNeasy Blood & Tissue Kit (Qiagen) according to manufacturer’s protocol. A library of total genomic DNA was prepared by the Australian Genome Research Facility (AGRF), Melbourne, and sequenced with 100 bp paired-end reads on an Illumina Hi-Seq 2000 platform (AGRF). Adapter sequences and low-quality bases were removed from the sequencing reads using Trimmomatic (Bolger et al. 2014). *De novo* assemblies were conducted with CLC Genomics Workbench 7.0 (CLCbio, Aarhus, Denmark) using default settings. Mitochondrial protein-coding and rRNA gene sequences of all published annelids were used as tblastn queries to search for mitochondrial fragments in the *Sa. spallanzanii* assembly. The top-hitting contig identified by BLASTN recovered the entire mitochondrial genome of *Sa. spallanzanii*. The final contig was annotated using MITOS server under the mitochondrial code for invertebrate mitochondria (Bernt et al. 2013), including the protein coding genes and the secondary structure of tRNAs and rRNAs. Gene boundaries generated from automatic annotations were manually examined and adjusted, and the tRNAs identified by MITOS were rechecked via the tRNAscan-SE web server (Schattner et al. 2005).

Amino-acid sequences of protein-coding genes of 14 annelids belonging to Sedentaria (as selected by Tilic et al. (2020b) from available mitochondrial genomes), including *M. occidentalis*, and an outgroup *Marphysa sanguinea* (Montagu 1813) belonging to Errantia, were used for phylogenetic analysis. Sequences were aligned using Muscle (Edgar 2004), implemented in Geneious 2020.0.5. The concatenated matrix was partitioned by gene. The best fitting evolutionary model for each partition was selected and the maximum likelihood analysis was performed in IQ-TREE 1.3.4 (Nguyen et al. 2015). Bootstrap support was estimated using an ultrafast bootstrap algorithm (UFBoot) (Minh et al. 2013) for 1000 replicates.

The mitochondrial genome of *Sa. spallanzanii* (GenBank accession number MW002660), length 15,581 bp, is AT-rich (64.5%), with an overall base composition of 34.4% (A), 30.1% (T), 12.3% (G) and 23.1% (C). The genome has negative GC-Skew (−0.31) and a positive AT-Skew (0.07). We identified 13 protein coding genes, 2 rRNAs and 22 tRNAs. Two copies of trnM were found. Two gene blocks ‘*cox1-cox2-atp8-trnK-cox3-trnD-trnE-trnW-trnV*’ and ‘*trnQ-trnA-trnY-trnM-trnL1-trnN-trnT*’ and gene *nad3* is found on the positive strand, while the other genes are transcribed from the negative strand. Encoding regions in the negative strand are unusual features of *Sa. spallanzanii*, because in other annelid mitochondrial genomes, all regions encode in the positive strand, except for some tRNAs in *Owenia fusiformis* Delle Chiaje, 1844, *Magelona mirabilis* (Johnston, 1865) (Weigert et al. 2016), and *Laeonereis culveri* (Webster, 1879); (Seixas et al. 2016).

The order of mitochondrial protein-coding genes and two rRNA genes in *Sa. spallanzanii* differs from the putative ground pattern (PGP) of Errantia and Sedentaria (Weigert et al. 2016) that is also observed in *M. occidentalis* (Tilic et al. 2020b). The genome of *Sa. spallanzanii* has three blocks: A–C. Block A (*cox1-cox2-atp8-cox3-nad6*) corresponds to the PGP. The gene orders in block B (*atp6-nad5-nad4l-nad4*) and C (*rrnS-rrnL-nad1*) in *Sa. spallanzanii* are reversed relative to the PGP (Figure 1). The *Sa. spallanzanii* mitogenome differs from that of *Sp. giganteus* where gene order is remarkably divergent from those of other annelid lineages (Seixas et al. 2017).

**Figure 1.**
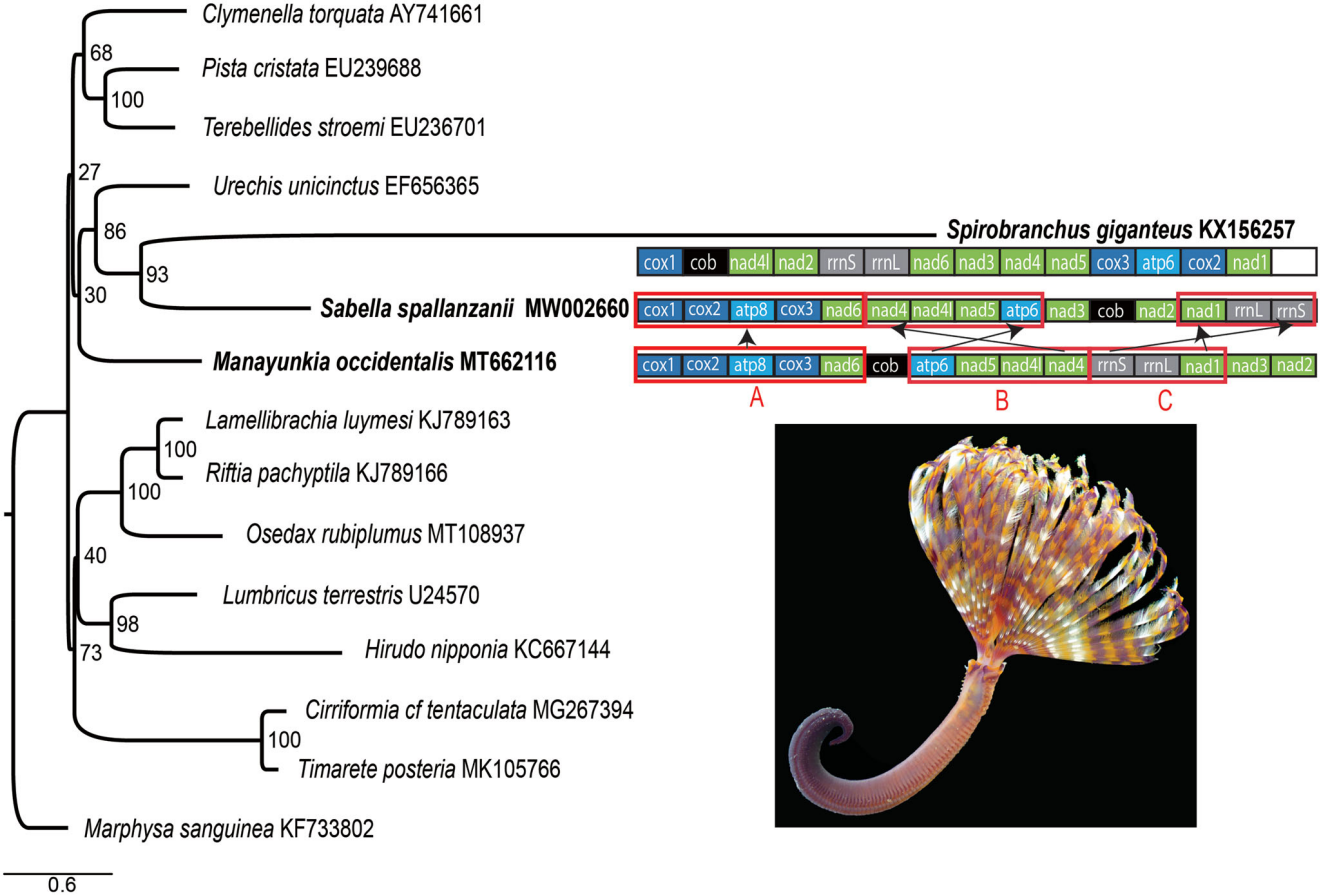
Maximum likelihood (ML) tree based on the concatenated amino acid sequences of mitochondrial protein-coding genes. Bootstrap support values are indicated at each node. *Marphysa sanguinea* (KF733802) was chosen as an outgroup. Gene order rearrangements in the Sabellida (Serpulidae + Sabellidae) relative to the annelid putative ground pattern (PGP) *sensu* Weigert et al. (2016) exemplified by *Manayunkia occidentalis*. Photo by S. Ahyong.

Maximum likelihood analyses recovered *Sa. spallanzanii* as sister group to *Sp. giganteus* (Figure 1). This placement supports the results of Tilic et al. (2020a), finding Sabellidae to be closer to Serpulidae than Fabriciidae. The Sabellidae + Serpulidae clade, however, did not group with *M. occidentalis*, but was recovered as sister to Echiura. Given that the high genetic distances between the mitogenome of *Sp. giganteus* and other annelids suggests rapid sequence evolution in serpulids (Seixas et al. 2017), the present result may be an artifact of rate heterogeneity on the long serpulid branch.

## Data Availability

The genome sequence data that support the findings of this study are openly available in GenBank of NCBI at https://www.ncbi.nlm.nih.gov/ under the accession no. MW002660. The associated BioProject number is PRJNA670556.

## References

[CIT0001] Ahyong ST, Kupriyanova E, Burghardt I, Sun Y, Hutchings PA, Capa M, Cox SL. 2017. Phylogeography of the invasive Mediterranean fan worm, *Sabella spallanzanii* (Gmelin, 1791), in Australia and New Zealand. J Mar Biol Ass. UK, 97(5):985–991.

[CIT0002] Atkinson SD, Bartholomew JL, Rouse GW. 2020. The invertebrate host of salmonid fish parasites *Ceratonova shasta* and *Parvicapsula minibicornis* (Cnidaria: Myxozoa), is a novel fabriciid annelid, *Manayunkia occidentalis* sp. nov. (Sabellida: Fabriciidae). Zootaxa. 4751(2):310–320. 10.11646/zootaxa.4751.2.6 32230420

[CIT0003] Bernt M, Donath A, Juhling F, Externbrink F, Florentz C, Fritzsch G, Putz J, Middendorf M, Stadler PF. 2013. MITOS: improved de novo metazoan mitochondrial genome annotation. Mol Phylogenet Evol. 69(2):313–319. 22982435 10.1016/j.ympev.2012.08.023

[CIT0004] Bolger AM, Lohse M, Usadel B. 2014. Trimmomatic: a flexible trimmer for Illumina sequence data. Bioinformatics. 30(15):2114–2120. 24695404 10.1093/bioinformatics/btu170 PMC4103590

[CIT0005] Clapin G, Evans DR. 1995. The status of the introduced marine fanworm *Sabella spallanzanii* in Western Australia: a preliminary investigation. Hobart, Australia: Centre for Research on Introduced Marine Pests, CSIRO Division of Fisheries. Technical Report 2. p. 1–34.

[CIT0006] Delle Chiaje S. 1844. Descrizione e Notomia degli Animali Invertebrati della Sicilia Citeriore osservati vivi negli anni 1822–1830. Tomo Ottavo. Appendice, Osservazioni Critiche, Indice Generale. Stabilamento Tipografico di C. Batelli e Comp., Napoli, 48. pp.

[CIT0007] Edgar RC. 2004. MUSCLE: multiple sequence alignment with high accuracy and high throughput. Nucleic Acids Res. 32(5):1792–1797. 15034147 10.1093/nar/gkh340 PMC390337

[CIT0008] Gmelin JF. 1791. Caroli a Linnaei Systema Naturae per Regna Tria Naturae, Edito Decima Tertia, Aucta, Reformata. Lipsiae. 1(6):3021–3910.

[CIT0009] Johnston G. 1865. A catalogue of the British non-parasitical worms in the collection of the British Museum. London: Trustees of the British Museum; p. 365.

[CIT0010] Montagu G. 1813. Descriptions of several new or rare animals, principally marine, found on the south coast of Devonshire. Trans Linn Soc Lond. 11(1):1–21.

[CIT0011] Minh BQ, Nguyen MAT, von Haeseler A. 2013. Ultrafast approximation for phylogenetic bootstrap. Mol Biol Evol. 30(5):1188–1195. 23418397 10.1093/molbev/mst024 PMC3670741

[CIT0012] Murray A, Keable SJ. 2013. First report of *Sabella spallanzanii* (Gmelin, 1791) (Annelida: Polychaeta) from Botany Bay, New South Wales, a northern range extension for the invasive species within Australia. Zootaxa. 3670(3):391–395. 26438950 10.11646/zootaxa.3670.3.10

[CIT0013] Nguyen LT, Schmidt HA, von Haeseler A, Minh BQ. 2015. IQ-TREE: a fast and effective stochastic algorithm for estimating maximum-likelihood phylogenies. Mol Biol Evol. 32(1):268–274. 25371430 10.1093/molbev/msu300 PMC4271533

[CIT0014] Pallas PS. 1766. *Miscellanea Zoologica*. Petrum van Cleef. The Hague: Hagae Comitum. 224pp.

[CIT0015] Read GB, Inglis G, Stratford P, Ahyong ST. 2011. Arrival of the alien fan worm *Sabella spallanzanii* (Gmelin, 1791) (Polychaeta: Sabellidae) in two New Zealand harbours. AI. 6(3):273–279.

[CIT0016] Schattner P, Brooks AN, Lowe TM. 2005. The tRNAscan-SE, snoscan and snoOPS web servers for the detection of tRNAs and snoRNAs. Nucleic Acids Res. 33(Web Server issue):686–689. 10.1093/nar/gki366 PMC1160127 15980563

[CIT0017] Seixas VC, Paiva PC, Russo CAM. 2016. Complete mitochondrial genomes are not necessarily more informative than individual mitochondrial genes to recover a well-established annelid phylogeny. Gene Rep. 5:10–17.

[CIT0018] Seixas VC, Russo CAM, Paiva PC. 2017. Mitochondrial genome of the Christmas tree worm *Spirobranchus giganteus* (Annelida: Serpulidae) reveals a high substitution rate among annelids. Gene. 605:43–53. 28034628 10.1016/j.gene.2016.12.024

[CIT0019] Tilic E, Sayyari E, Stiller J, Mirarab S, Rouse GW. 2020a. More is needed- Thousands of loci are required to elucidate the relationships of the ‘flowers of the sea’ (Sabellida, Annelida)). Mol Phylogenet Evol. 151:106892. 32562819 10.1016/j.ympev.2020.106892

[CIT0020] Tilic E, Atkinson SD, Rouse GW. 2020b. Mitochondrial genome of the freshwater annelid *Manayunkia occidentalis* (Sabellida: Fabriciidae). Mitochondrial DNA Part B. 5(3):3295–3315. 33458144 10.1080/23802359.2020.1815604 PMC7782465

[CIT0021] Webster HE. 1879. The Annelida Chaetopoda of New Jersey. Annu Rep N Y State Museum Nat His. 32:101–128.

[CIT0022] Weigert A, Golombek A, Gerth M, Schwarz F, Struck TH, Bleidorn C. 2016. Evolution of mitochondrial gene order in Annelida. Mol Phylogenet Evol. 94(Pt A):196–206. 26299879 10.1016/j.ympev.2015.08.008

